# Supplementary material for: Hyaluronan and Collagen Are Prominent Extracellular Matrix Components in Bovine and Porcine Ovaries
Source: Genes (Basel). 2021 Jul 30;12(8):1186. doi: 10.3390/genes12081186 (PMC8392036; doi:10.3390/genes12081186)
Supplement: Supplementary file 1 [file genes-12-01186-s001.zip › genes-1267849-supplementary_Updated.pdf]

genes

MDPI

Supplemental Material

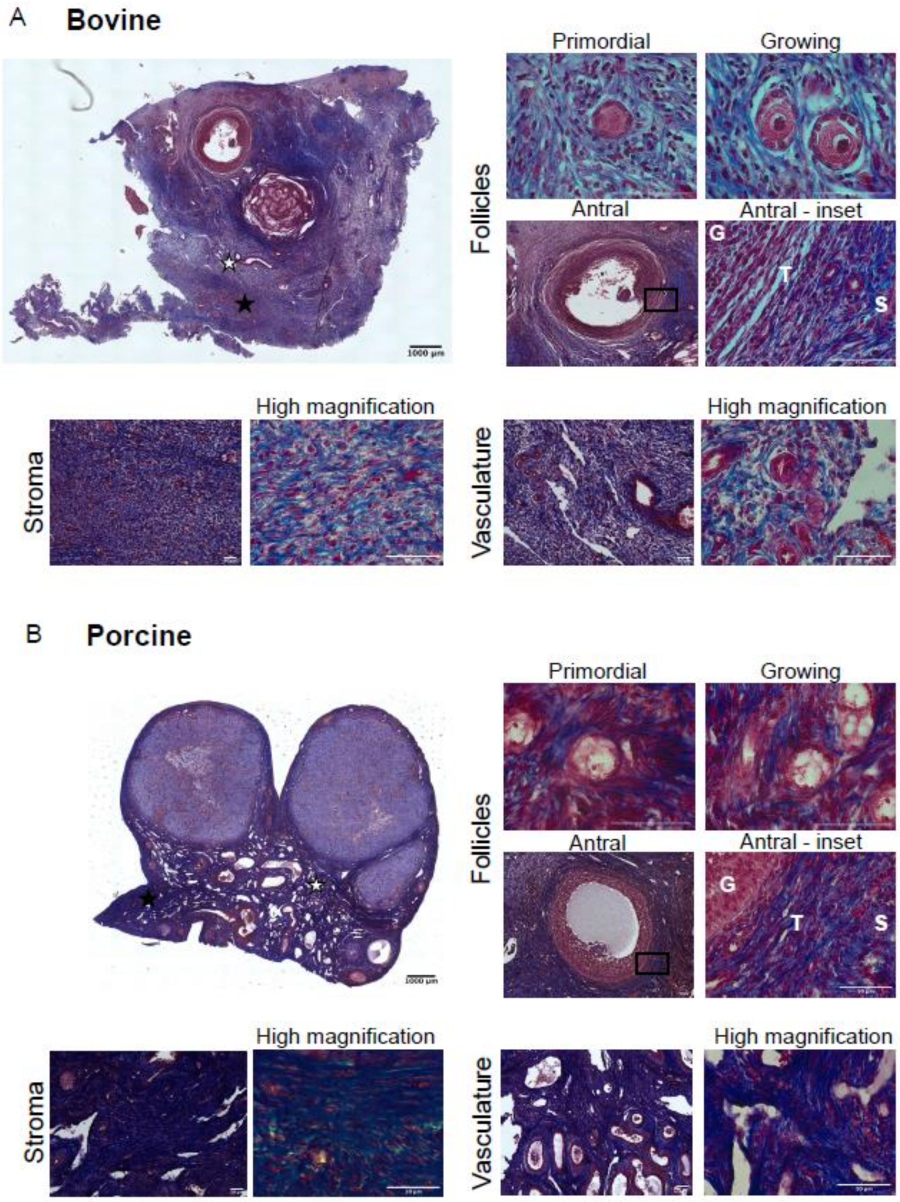

Figure 1. Bovine and porcine ovaries are enriched in collagen. Masson's trichrome (MTC) staining was used to detect collagen (blue) in (A) bovine or (B) porcine ovaries. In the low magnification whole tissue section images of both species, black stars indicate the location of the stroma and white stars indicate the location of the vasculature. Collagen was found around primordial follicles, growing follicles, and antral follicles of both species. The high magnification porcine antral follicle image was taken from a different ovarian tissue section. S – stroma, T – theca cell layer, G – granulosa cell layer. The scale bars are in 1000 μm in the low magnification whole ovarian tissue section images, 10 μm in the primordial and growing follicle images, and 50 μm in the antral follicle, stroma, and vasculature images. MTC Images are representative of n = 2 cows, n = 1 ovary/cow, 2 sections/ovary or n = 2 pigs, n = 2 ovaries/pig, 2 sections/ovary.

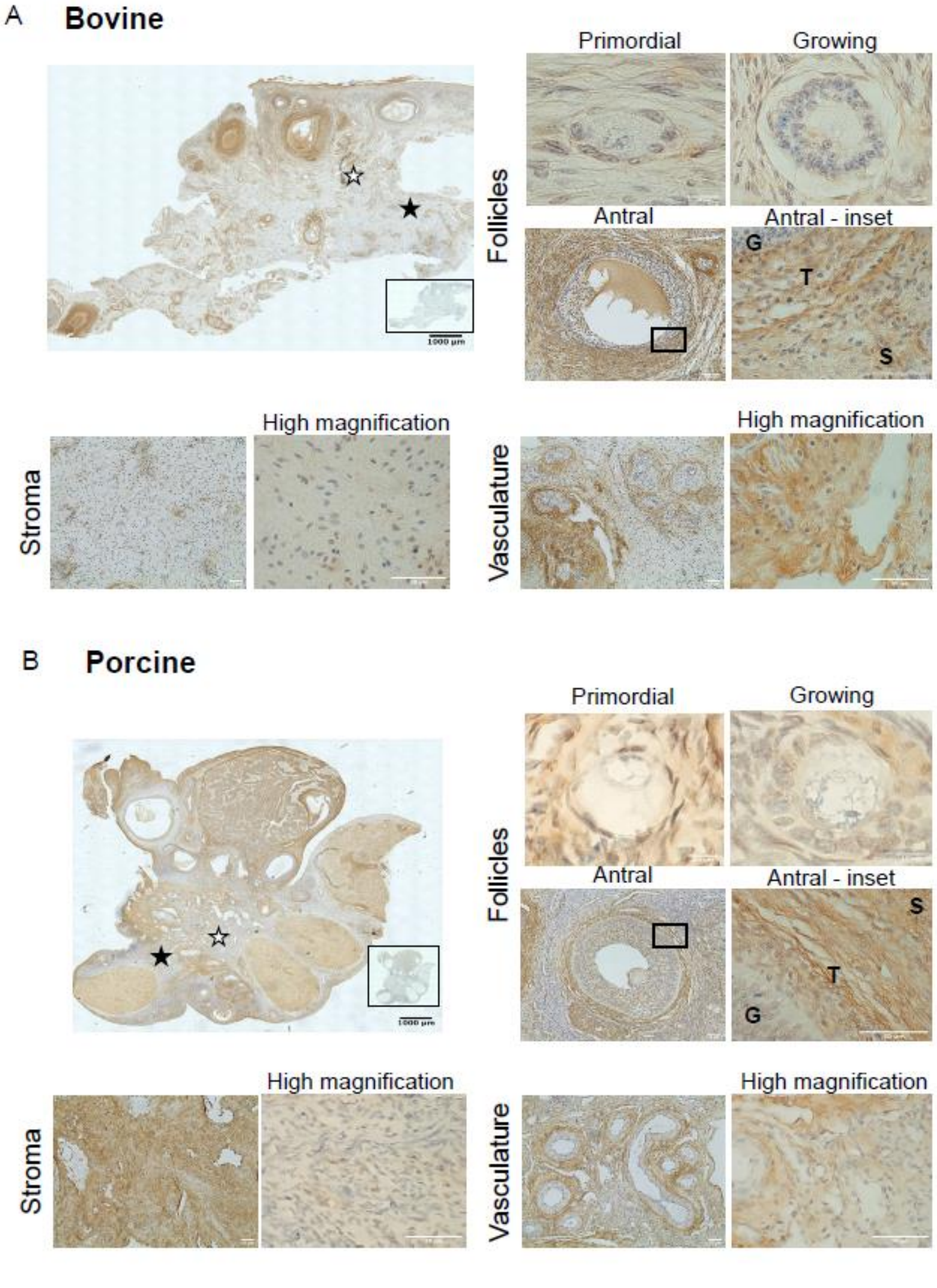

Figure 2. Bovine and porcine ovaries are enriched in hyaluronan. Chromogenic HABP staining was used to detect HA (brown) in (**A**) bovine or (**B**) porcine ovaries. The insets illustrate HABP staining after pretreating a serial bovine or porcine ovarian tissue section with hyaluronidase. In the low magnification whole tissue section images of both species, black stars indicate the location of the stroma and white stars indicate the location of the vasculature. HA was identified in primordial, growing, and the antral follicles. The high magnification porcine antral follicle image was taken from a different ovarian tissue section. S – stroma, T – theca cell layer, G – granulosa cell layer. The scale bars are in 1000 µm in the low magnification whole ovarian tissue section images, 10 µm in the primordial and growing follicle images, and 50 µm in the antral follicle, stroma, and vasculature images. Images are representative of n = 2 cows, n =1 ovary/cow, n = 2 sections/ovary and n = 2 pigs, n = 2 ovaries/pig, n = 2 sections/ovary.

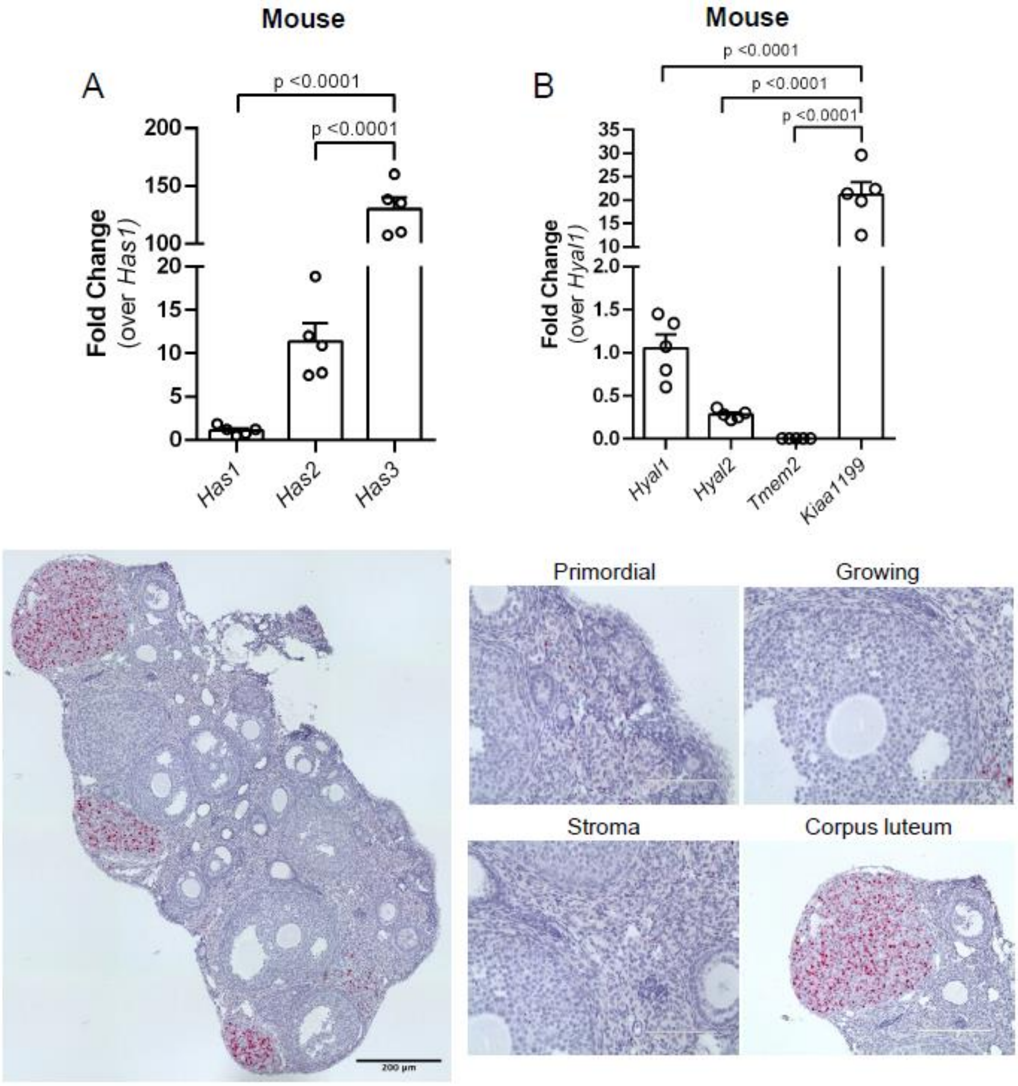

Figure 3. Mouse ovary hyaluronan synthase and hyaluronidase transcript content. Relative hyaluronan synthase (**A**) and hyaluronidase (**B**) gene expression in mouse ovarian stromal tissue. Hyaluronan synthase transcript data are shown as fold change over *Has1*, while hyaluronidase transcript data are shown as a fold change over *Hyal1*, $n = 5$ mice, n = 1 ovary/mouse. Circles represent values from individual animals. Statistical analysis was performed using one-way ANOVA. p-values are as indicated. (**C**) *Kiaa1199* transcripts (pink) were identified in a whole mouse ovarian tissue section, in primordial and growing follicles, and in ovarian stroma and corpus luteum using RNA *in situ* hybridization. The scale bars are in 200 µm in the low magnification whole mouse ovarian tissue section, 100 µm in the primordial and growing follicle images and in the stroma images, and 200 µm in the corpus luteum image. Images are representative of $n = 2$ mice, $n = 1$ ovary/mouse, $n = 3$ sections/mouse.

Supplementary Table S1: Primers and primer/probe assays used for RT-PCR

Species	Company	Chemistry	Unique assay ID	Gene	Region	Sequences
Cow	Invitrogen	TaqMan Assay	Bt07109172_g1	*Has1*	Both primers and probe map within a single exon	Proprietary
Cow	Invitrogen	TaqMan Assay	Bt03212695_g1	*Has2*	Probe spans exons	Proprietary
Cow	Invitrogen	TaqMan Assay	Bt04298491_m1	*Has3*	Probe spans exons	Proprietary
Cow	Invitrogen	TaqMan Assay	Bt03219822_m1	*Hyal1*	Probe spans exons	Proprietary
Cow	Invitrogen	TaqMan Assay	Bt03223415_m1	*Hyal2*	Probe spans exons	Proprietary
Cow	Invitrogen	TaqMan Assay	Bt04312260_m1	*Tmem2*	Probe spans exons	Proprietary
Cow	Invitrogen	TaqMan Assay	Bt03256165_m1	*Gusb*	Probe spans exon	Proprietary
Cow	Bio-Rad	Prime PCR SYBR Green Assay	qBtaCED0009350	*Col1a1*	Exonic	Proprietary
Cow	Bio-Rad	Prime PCR SYBR Green Assay	qBtaCED0017751	*Col3a1*	Exonic	Proprietary
Cow	Bio-Rad	Prime PCR SYBR Green Assay	qBtaCID0004725	*Gusb*	Intron spanning	Proprietary
Pig	Bio-Rad	Prime PCR Probe Assay	qSscCEP0032506	*Has1*	Exonic	Proprietary
Pig	Bio-Rad	Prime PCR Probe Assay	qSscCEP0030548	*Has2*	Exonic	Proprietary
Pig	Bio-Rad	Prime PCR Probe Assay	qSscCEP0032789	*Hyal1*	Exonic	Proprietary
Pig	Bio-Rad	Prime PCR Probe Assay	qSscCEP0032796	*Hyal2*	Exonic	Proprietary
Pig	Bio-Rad	Prime PCR Probe Assay	qSscCIP0027390	*Kiaa1199*	Intron spanning	Proprietary
Pig	Bio-Rad	Prime PCR Probe Assay	qSscCIP0034945	*Gusb*	Intron spanning	Proprietary
Pig	Bio-Rad	Prime PCR SYBR Green Assay	qSscCED0020342	*Col1a1*	Exonic	Proprietary
Pig	Bio-Rad	Prime PCR SYBR Green Assay	qSscCID0003467	*Col3a1*	Intron spanning	Proprietary
Pig	Bio-Rad	Prime PCR SYBR Green Assay	qSscCID0013585	*Gusb*	Intron spanning	Proprietary
Mouse	Invitrogen	TaqMan Assay	Mm03048195_m1	*Has1*	Probe spans exons	Proprietary
Mouse	Invitrogen	TaqMan Assay	Mm00515089_m1	*Has2*	Probe spans exons	Proprietary
Mouse	Invitrogen	TaqMan Assay	Mm00515092_m1	*Has3*	Probe spans exons	Proprietary
Mouse	Invitrogen	TaqMan Assay	Mm01197698_m1	*Gusb*	Probe spans exons	Proprietary
Mouse	Integrated DNA Technologies	Power SYBR Green (Invitrogen)	PrimerBank ID: 145966880b3	*Hyal1*	Exonic	F: TTCGATGTGGTTGCCAACAAG R: AGGTGCCCAATTCCTCTCTGT
Mouse	Integrated DNA Technologies	SYBR Green	PrimerBank ID: 45331201b1	*Hyal2*	Intron spanning	F: GCAGGACTAGGTCCCATCATC R: TTCCATGCTACCACAAAGGGT
Mouse	Integrated DNA Technologies	SYBR Green	Designed by Dr. Chris Ward, using XM_006527487.3	*Tmem2*	Intron spanning	F: CTTTACCTTCCGGAGTGCAG

			(now NM_031997.4)			R: CCGCTGAATCCCAAAAATAC
Mouse	Integrated DNA Technologies	SYBR Green	NM_030728.4	*Kiaa1199*	Intron spanning	F: TGATGGGAGTCGAGGTCAC R: GAGCACTATGGAATTGTCAGG
Mouse	Integrated DNA Technologies	SYBR Green	PrimerBank ID: 6754098a1	*Gusb*	Intron spanning	F: GGCTGGTGACCTACTGGATTT R: GGCACTGGGAACCTGAAGT
Mouse	Integrated DNA Technologies	SYBR Green	World J. Gastro. 2012 4(12):356	*Col1a1*	Intron spanning	F: ATGTTCAGCTTTGTGGACCTC R: CAGAAAGCACAGCACTCGC
Mouse	Integrated DNA Technologies	SYBR Green	PrimerBank ID: 33859526a1	*Col3a1*	Exonic	F: ACGTAGATGAATTGGGATGCAG R: GGGTTGGGGCAGTCTAGTG
Goat *Cow	Invitrogen	TaqMan Assay	Ch04792899_m1	*Kiaa1199*	Probe spans exons	Proprietary
Human *Pig	Bio-Rad	Prime PCR Probe Assay	qHsaCIP0026619	*Has3*	Intron spanning	Proprietary
Human *Pig	Bio-Rad	Prime PCR Probe Assay	qHsaCIP0032990	*Tmem2*	Intron spanning	Proprietary

*For cow *Kiaa1199*, pig *Has3*, and pig *Tmem2*, goat *Kiaa1199*, human *Has3*, and human *Tmem2* Prime PCR assays, respectively, were used as the primer and probe sequences for the paired species were identical (per Bio-Rad).
